# Multifocal tuberculosis-associated immune reconstitution inflammatory syndrome – a case report of a complicated scenario

**DOI:** 10.1186/s12879-019-4182-1

**Published:** 2019-06-17

**Authors:** Gopalan Narendran, Deivide Oliveira-de-Souza, Caian L. Vinhaes, Kevan Akrami, Kiyoshi F. Fukutani, Kesavamurthy Banu, Padmapriyadarsini Chandrasekaran, Narayanan Ravichandran, Irini Sereti, Soumya Swaminathan, Bruno B. Andrade

**Affiliations:** 1 National Institute for Research in Tuberculosis, Chennai, 600031 India; 2 Instituto GonçaloMoniz, Fundação Oswaldo Cruz, Salvador, 40296-710 Brazil; 3 Multinational Organization Network SponsoringTranslationaland Epidemiological Research (MONSTER) Initiative, Fundação José Silveira, Salvador, 40210-320 Brazil; 4 Curso de Medicina, Faculdade de Tecnologia e Ciências (FTC), Salvador, 40290-150 Brazil; 5 Division of Infectious Diseases, Department of Medicine, University of California, San Diego, USA; 6 Institute of Neurology, Madras Medical College, Chennai, India; 7 Government Hospital of Thoracic Medicine, Tambaram, Chennai, India; 8 National Institutes of Allergy and Infectious Diseases, National Institutes of Health, Bethesda, MD USA; 9 Wellcome Centre for Infectious Disease Research in Africa, Institute of Infectious Disease and Molecular Medicine, University of Cape Town, Cape Town, 7925 South Africa; 10 Division of Infectious Diseases, Department of Medicine, Vanderbilt University School of Medicine, Nashville, TN 37232 USA; 11 Universidade Salvador (UNIFACS), Laureate Universities, Salvador, 41720-200 Brazil; 12 Escola Bahiana de Medicina e Saúde Pública (EBMSP), Salvador, 40290-000 Brazil

**Keywords:** Immune reconstitution inflammatory syndrome, IRIS, Tuberculosis, HIV

## Abstract

**Background:**

Tuberculosis (TB)-associated Immune reconstitution inflammatory syndrome (TB-IRIS) is an aberrant inflammatory response in TB patients with advanced human immunodeficiency virus coinfection, after antiretroviral therapy commencement.

**Case presentation:**

We present a rare case of a 51-year-old woman living with HIV who developed a series of TB-IRIS events occurring at multiple sites sequentially, highlighting the clinical complexity in diagnosis and management.

**Conclusion:**

This case illustrates how complicated a clinical scenario of successive TB-IRIS episodes can be, in terms of clinical management.

## Background

Immune reconstitution inflammatory syndrome (IRIS) poses important challenges in the clinical management of HIV-TB patients initiating antiretroviral therapy (ART). In HIV patients with TB, IRIS is thought to occur in two distinct scenarios. First, unmasking of a previously undiagnosed TB infection prior to ART initiation. Alternatively, paradoxical clinical or radiological worsening of TB in patients previously improving with antituberculous treatment (ATT) following ART introduction and despite effective virological suppression [1]. The pathogenesis of IRIS remains incompletely understood. The most significant event leading to TB-IRIS in HIV is failure of the immune system to eliminate *Mycobacterium tuberculosis*, causing high mycobacterial burden in those with severe immunosuppression and lymphopenia prior to ART, followed by its restitution and immune activation [2]. Contrary to the belief that IRIS is a rare condition, a recent meta-analysis demonstrated a pooled estimated incidence of up to 18% among HIV-associated TB initiating ART [3]. Here, we present a rare case of both unmasking and paradoxical TB-IRIS occurring at multiple sites sequentially, highlighting the clinical complexity in diagnosis and management.

## Case presentation

**Fig. 1:**
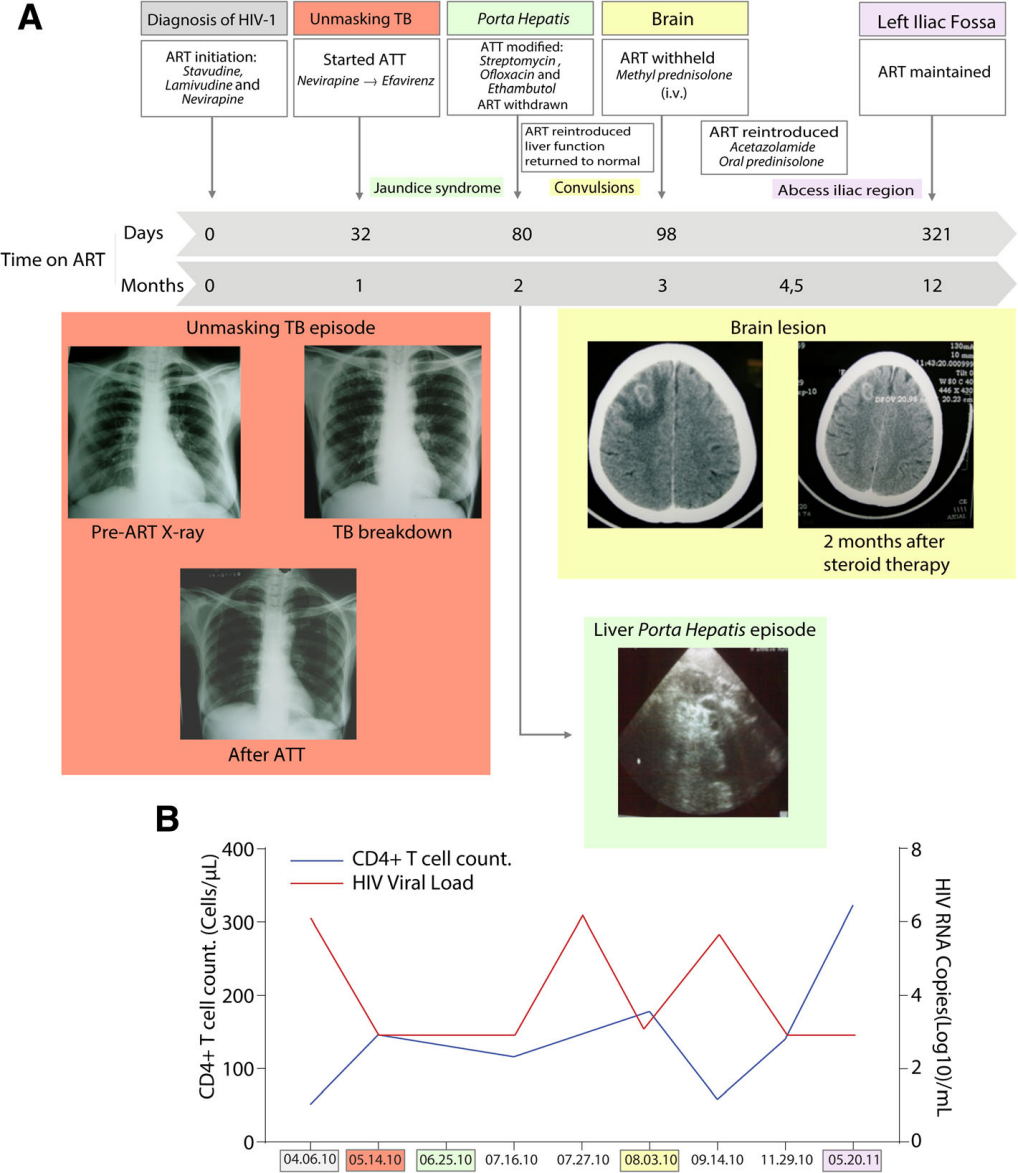
Multifocal TB-IRIS: timeline of events. **a** Timeline of the clinical evolution show the temporal relationship between IRIS events with time on ART and ATT. Colored boxes indicated distinct clinical events with the respective radiological examination. **b** CD4+ T-cell count and viral load in each IRIS clinical episode

An asymptomatic 51-year-old woman without significant past medical history was diagnosed with HIV-1 infection on April 06, 2010 after her spouse died of HIV. Her baseline Chest X-ray was reported as normal and physical examination did not revealed any relevant clinical signs. She presented with a CD4^+^ T-cell count of 51 cells/μL and HIV viral load of 5.8 log_10_ copies/mL. ART was initiated with stavudine, lamivudine and nevirapine as per Indian National guidelines [4]. After 32 days on ART, she presented a clinical deterioration, with cough, afternoon fever, weight loss and night sweat, symptoms suggestive of pulmonary TB and subsequently confirmed to be drug-sensitive *M. tuberculosis* by sputum smear and culture (Fig. 1a). Standard ATT was started with Isoniazid, Rifampicin, Ethambutol, Pyrazinamide on May 08, 2010 (Fig. 1a) with nevirapine substituted for efavirenz. Repeat laboratory results revealed a CD4^+^ T-cell count of 146 cells/μL and viral load of < 2log_10_ copies/mL (400 copies/mL) (Fig. 1b). A panel of independent physicians reviewed the patient’s history, radiographs and physical examination. This independent panel of clinicians used the INSHI definition of unmasking TB IRIS [5], composed by the following criteria: not receiving TB treatment at ART initiation; diagnosis of active TB after ART initiation; fulfilling WHO diagnostic criteria for TB; presentation within 3 months of ART initiation and heightened intensity of clinical manifestations once on TB treatment. The panel concluded that the patient had unmasking TB-IRIS at ART initiation.

After 48 days since ATT and efavirenz based ART were initiated, the patient presented with generalized pruritus and strong vague abdominal pain. Physical examination revealed fever, jaundice and left sided cervical lymphadenopathy. Laboratory tests were notable for high levels of total bilirubin 8.3 mg/dL (reference value [RV]: 0.2–1.0 mg/dL) with increases in aspartate aminotransferase AST, 72 U/L (RV: 5.0–40.0 U/L) and alanine aminotransferase ALT, 59 U/L (RV: 7.0–56.0 U/L). Abdominal ultrasound revealed enlargement and obstruction of the *porta hepatis* by periportal nodes with normal echotexture of the liver and gall bladder. At this crisis, ATT was modified to streptomycin, ofloxacin and ethambutol, second-line therapy (Fig. 1a), and ART was temporarily withheld, following advice from the panel of physicians reviewed, due to potential ART-induced liver toxicity. On July 19, 2010, with the liver function tests returning to normal (Table 1), standard ATT regimen was re-introduced. After 11 days, ART was reinitiated as the HIV viral load was > 5.9log_10_copies/mL (Fig. 1b).

**Table 1:** Plasma measurements according to date of assessment

Date	05.14.10	06.25.10	07.16.10	07.27.10	08.03.10	09.14.10	11.29.10	5.29.11
Hb (g/dL)	7.6		10.7		10.2	11.3	12.2	13.3
RBC (Cells/mm^3^)	2.93		3.75		3.47	4.15	4.32	4.15
WBC (Cells/mm^3^)	10,600		5600		7900	9100	8700	9200
Platelets(× 10^3^/mm^3^)	543		365		464	285	386	544
CD4 (Cells/mm^3^)	146		116		178	58	140	323
CD8(Cells/mm^3^)	386		662		441	593	778	1466
Viral Load (Log_10_copies/mL)	< 2			> 6	2.83	5.3	< 2	< 2
Direct Bilirubin (mg/dL)	0.6	8.3	2	2.7	1.2		0.4	0.4
AST (IU/L)	65	72	36	125	30		29	27
ALT (IU/L)	63	59	27	61	23		22	21

*ALT* alanine aminotransferase, *AST* aspartate aminotransferase, *Hb* hemoglobin, *RBC* red blood cells, *WBC* white blood cells

One week after ART reintroduction, the patient developed rapid clinical deterioration with focal complex partial convulsions prompting initiation of anticonvulsant therapy. Brain Computational Tomography (CT) scan revealed a frontoparietal space occupying lesion with peri-lesional, edema suggestive of tuberculoma (Fig. 1a). Following the CT scan results, cerebral spinal fluid was collected, and results excluded other potential opportunistic infections, such as neurocryptococcosis or toxoplasmosis. ART was again withheld temporarily while continuing ATT, because the independent clinical panel suggested the possibility of severe central nervous system (CNS) IRIS. Initially, she was treated with intravenous steroids and mannitol followed by acetazolamide to reduce perilesional edema. Oral prednisolone was gradually tapered over 8 weeks. Laboratory investigations demonstrated CD4^+^ T-cell count of 178cells/μL and HIV viral load of 2.83 log_10_ copies/mL. The patient recovered after 6 weeks, only to have a viral rebound (HIV viral load of 5.3 log_10_ copies/mL) (Fig. 1b). It was decided to reintroduce ART as an inpatient with stringent clinical monitoring while on oral steroids (Fig. 1a). In November 29, 2010, a repeat brain CT scan showed regression and calcification of the lesion. ATT was stopped on December 6, 2010 (total of 8-month duration) while ART and anticonvulsants were continued based on a consensus opinion from IRIS experts, TB experts and neurologists.

In May 2011, 5 months after completing ATT, the patient developed a swelling in the left iliac region. X-ray and CT of the spine were normal while ultrasonogram identified a localized intramuscular abscess which was drained under guidance, and the aspirate was negative for acid-fast bacilli (AFB) in smear and cultures for mycobacteria and gram positive or negative bacteria, as well as fungi. Laboratory tests results showed CD4^+^ T-cell count of 323 cells/μL and HIV viral load < 2 log_10_ copies/mL (Fig. 1b). Other laboratory exams were normal (Table 1). Repeat CT scan of the brain showed further regression and calcification of the tuberculoma. Thereafter, the patient remained clinically asymptomatic and was followed for 30 months. Smears and cultures 30 months post ATT treatment were all negative after she converted in the first month and viral loads were suppressed as well.

## Discussion and conclusion

TB-IRIS is an exaggerated, dysregulated response against *M. tuberculosis* that frequently occurs after initiation of ART despite effective suppression of HIV viremia and a temporal improvement with ATT [6]. TB-IRIS is a common phenomenon in HIV-TB coinfected patients, ranging from 2% [7] to 54% [8] depending on predisposing factors, such as the degree of immunological suppression prior to ART [2] and the presence of disseminated disease. It is thought that poor immunity against *M. tuberculosis* in highly immunosuppressed HIV+ patients results in delayed clearance of the opportunistic pathogen leading to elevated mycobacterial load and dissemination of infection [1]. Once there is CD4+ T-cell recovery, followed by restoration of immune responses, hyper activation of infected macrophages and dendritic cells leads to increased production and release of pro-inflammatory mediators such as interleukin (IL)-1β, IL-6 and IL-18 at infection sites [1]. Such hypercytokinaemia [9] is a hallmark of TB-IRIS. The case presented here exhibited multiple IRIS episodes, or late TB-IRIS onset [10, 11] which posed challenges in clinical management. Multifocal TB-IRIS is uncommon. Our main hypothesis is that our patient was highly immunosuppressed as reflected by her CD4+ T-cell count, and thus had mycobacterial dissemination to several different sites. We have recently reported that presence of extrapulmonary TB is linked to increased odds of TB-IRIS in a South Indian cohort, but a logistic regression model adjusted for other factors such as CD4+ T-cell counts and HIV viral load resulted in loss of significance [12], probably because these variables are highly associated with each other. In the case presented here, the restoration of immunity following ART caused a series of inflammatory perturbations with accompanying episodes of clinical deterioration occurring in specific infection sites. Compartmentalization of immune responses/reconstitution could also explain the distinct events affecting different locations.

The bacillary loads and risk of infection dissemination are usually high in patients with advanced HIV infection [13]. Perhaps for this reason the patient could have initially presented with unmasking pulmonary TB. The hepatic portal event, occurring in the first 4 weeks, may have also been a consequence of rapid immune-mediated mycobacterial clearance associated with a high bacillary load at the affected site prior to immune reconstitution. With ATT modification and ART withdraw, immune recovery decreased its pace and liver profile was restored. The new neurological presentation after re-initiation of ART could be caused by at least two distinct mechanisms: (i) there was a HIV viral load rebound subsequent to stopping ART and then re-starting ART, which could have increased risk of paradoxical TB-IRIS-like phenomenon; (ii) it is possible that the revised TB regimen after the hepatic porta event had poor central nervous system penetration and that for this reason disseminated TB has progressed at this site. With reestablishment of high microbial burden at the time of ART reintroduction, again an exaggerated and dysregulated immune response occurred, and at this time in the brain, leading to focal seizures with CT imaging consistent with tuberculoma. This latter event had a critical and rapid deterioration of clinical symptoms (severe IRIS episode), leading to a new withdraw of ART. Again, with ART suspended, the exaggerated response declined, allowing clinical stability. Lastly, several months after ATT, the patient probably had cleared or highly reduced mycobacterial loads, which potentially were still not able to prevent occurrence of the last episode of abscess in the left iliac fossa. This case illustrates how complicated a clinical scenario of successive TB-IRIS episodes can be, in terms of clinical management. The complexity associated with rapid clinical deterioration, and oscillation of clinical status between IRIS episodes, makes the therapeutic management a relevant challenge. The ART interruption, observed two times during case development, maybe have had influenced mycobacterial dissemination and multifocal IRIS presentation.

Given the current WHO policy of test and treat with earlier ART initiation [14], incidence of IRIS may decrease as individuals will be treated earlier and with higher CD4+ T-cell counts. This case demonstrates the intricate challenges in managing of highly immunosuppressed patients with TB-HIV co-infection at the time of ART commencement, and emphasizes discussion about several relevant aspects presented here, such as: (i) risks associated with ART interruption during ATT in the context of advanced HIV disease, (ii) the potential hepatotoxicity of combined ATT and ART therapy and (iii) reduced penetration of the drugs in organs/tissues and its potential association with occurrence of IRIS. These discussions call attention of the clinical community to the several nuances in IRIS management.

## Data Availability

All data containing relevant information to support the study findings are provided in the manuscript.

## Abbreviations

AFB: Acid-fast bacilli
ART: Antiretroviral therapy
ATT: Antitubercular therapy
CNS: Central nervous system
IRIS: Immune Reconstitution Inflammatory Syndrome
